# Genetic Dissection of ICP-Detected Nutrient Accumulation in the Whole Seed of Common Bean (*Phaseolus vulgaris* L.)

**DOI:** 10.3389/fpls.2016.00219

**Published:** 2016-03-08

**Authors:** Matthew Wohlgemuth Blair, Xingbo Wu, Devendra Bhandari, Carolina Astudillo

**Affiliations:** ^1^Department of Agricultural and Environmental Sciences, Tennessee State University, NashvilleTN, USA; ^2^Bayer Crops Research, DavisCA, USA

**Keywords:** dry bean grain, inductively coupled plasma spectroscopy, mineral and non-mineral elements, pulse legume, quantitative trait loci, seed nutrient concentration

## Abstract

Nutrient transport to grain legume seeds is not well studied and can benefit from modern methods of elemental analysis including spectroscopic techniques. Some cations such as potassium (K) and magnesium (Mg) are needed for plant physiological purposes. Meanwhile, some minerals such as copper (Cu), iron (Fe), molybdenum (Mo), and zinc (Zn) are important micronutrients. Phosphorus (P) is rich in legumes, while sulfur (S) concentration is related to essential amino acids. In this research, the goal was to analyze a genetic mapping population of common bean (*Phaseolus vulgaris L.*) with inductively coupled plasma (ICP) spectrophotometry to determine concentrations of and to discover quantitative trait loci (QTL) for 15 elements in ground flour of whole seeds. The population was grown in randomized complete block design experiments that had been used before to analyze Fe and Zn. A total of 21 QTL were identified for nine additional elements, of which four QTL were found for Cu followed by three each for Mg, Mn, and P. Fewer QTL were found for K, Na and S. Boron (B) and calcium (Ca) had only one QTL each. The utility of the QTL for breeding adaptation to element deficient soils and association with previously discovered nutritional loci are discussed.

## Introduction

Legumes are nutrient rich sources of many essential minerals and trace elements needed for human health and plant growth (Grusak and DellaPenna, 1999). While most research has concentrated on iron (Fe) and zinc (Zn) accumulation for legume biofortification programs (reviewed in Bouis, 2003; Wang et al., 2003; Welch and Graham, 2004; Blair, 2013a), other nutrients are necessary elements for plant physiological purposes and for the human diet. These include boron (B), calcium (Ca), copper (Cu), manganese (Mn), magnesium (Mg), potassium (K), molybdenum (Mo), sodium (Na), and sulfur (S) (White and Broadley, 2009).

While intake of major nutrients such Ca, Mg, K, and Na is generally assumed to be easily met by a fiber intake and a protein-adequate diet, some trace elements like B, Cu, and Zn are often deficient in the diet (Welch and Graham, 1999). Although abundant in nature, Fe is difficult to absorb because in most soils it is found in the oxidized form that is less available to plants and consequently animals, causing iron deficiency anemia (Frossard et al., 2000; Welch, 2002). Zn is rare but is an integral part of many enzyme systems, including DNA polymerase, such that Zn deficiency causes stunted growth and delayed sexual development (Jeejeebhoy, 2009). B is required by plants for seedling development but also by humans for bone growth and central nervous system function (Branca and Ferrari, 2002; Nielsen, 2014). Cu is a centerpiece of reducing anti-oxidative stress (Gaetke and Chow, 2003). Bone development and bone mass in humans are associated with Ca, whose intake in adequate amounts prevents fractures and osteoporosis (Nordin, 1997; Martínez et al., 2002a; Bass and Chan, 2006; Rizzoli et al., 2008). Similarly, K and Na are involved in all human tissues for ion transport across membranes; P and Mg are involved in cellular production of adenosine triphosphates (ATP); while S is critical to the biosynthesis of essential amino acids (Martínez et al., 2002b; Métayer et al., 2008).

Minerals in bean seeds can be analyzed individually by atomic absorption or in parallel by inductive coupled plasma (ICP) or near infrared reflectance (NIR) methods (Blair, 2013a). The advantage of ICP as a modern chemical analytical technique is that the method can analyze a suite a minerals all together as a set rather than individually. Inductively coupled plasma (ICP) optical emission spectrometry (ICP-OES) or atomic emission spectroscopy (ICP-AES) abbreviated ICP is a powerful tool to evaluate many elements from the periodic table simultaneously in a single assay often using multiple scans for precise and rapid data collection. Typically 10–16 elements are evaluated at once, however, up to 32 elements can be measured in one assay (Cubadda et al., 2002). The elements vary in their detection level thresholds with most detectable in parts per million (ppm) up to parts per trillion (ppt).

Both metal and non-metal elements can be analyzed by ICP-OES; however, mainly a few trace minerals and important cations are emphasized in agricultural applications especially those related to plant tissue analysis. The technique has been used for iron and zinc concentration evaluations in grain legumes such as common beans, *Phaseolus vulgaris* (Blair et al., 2009a, 2010a,b, 2011, 2012, 2013), garden peas, *Pisum sativum* (Cheng et al., 2015) and peanuts, *Arachis* (Phan-Thien et al., 2012) as well as cereals (Cakmak et al., 2010). In the case of peas a full spectrum of over 12 minerals has been analyzed on two germplasm collections (Cheng et al., 2015). Up to 32 elements can be analyzed by ICP and next generation studies are likely to report a fuller gamut of minerals. Other samples analyzed with ICP methods include tobacco and cabbage leaves (Lachas et al., 2000) as well as meat and seafood (Cubadda et al., 2002). ICP can also be used to measure toxic heavy metals in plants, including cadmium (Cd), cobalt (Co), and nickel (Ni) from irrigated areas where they tend to accumulate to high concentrations causing environmental concerns for plants and people (Muto et al., 1994).

The mechanism of element analysis in ICP is based on an ion source, an analytical nebulizer into a sample carrier gas, a quartz torch for creating high temperature argon plasma, an induction coil for conducting the plasma to a series of cones, and a multi-spectral detector for measuring the elements in the resulting ionized samples (Ammann, 2007). The first step for the analysis is the creation of an aerosolized liquid sample of the tissue being analyzed followed by disassociation of the molecules from their surrounding matrix using a very high temperature in theargon plasma. The charged ions with characteristic wavelengths are then detected by various types of optical spectrometers depending on the ICP machines design (Houk et al., 1980). The small-droplet, aerosolized samples can be injected by a peristaltic pump into the chamber; or alternatively, the liquid samples can be nebulized after aspiration into the chamber. These are exposed to the high energy plasma source and then analyzed for elemental concentrations by special software correcting for interference by different elements within the sample (Łobiński et al., 2006).

The ICP method compares favorably to electrospray ionization mass spectroscopy (ESI-MS) and matrix assisted laser desorption/ionization mass spectroscopy (MALDI-MS), two other types of ion separation which are used for complex biomolecules such as proteins, peptides, lipids, and other small molecules rather than simple minerals and elemental constituents of a plant tissue sample (Caruso et al., 2000). Some methods of mass spectroscopy also provide element and isotopic information but quantification is difficult compared to ICP (Łobiński et al., 2006). The ICP method provides a holistic approach to multi-elemental composition providing total content of a large number of elements being high throughput and precise in quantification (Łobiński et al., 2006). Due to its high information gathering ability, the ICP method is a low cost technique although argon gas is an expensive carrier used by most ICP-OES machines.

The purpose of this study was to use the ICP-OES technique to evaluate the accumulation of minerals and elements in common bean seeds and to genetically dissect the quantitative trait loci (QTL) controlling these traits that are of interest to human health and plant physiology. Specific objectives were to (1) determine the range of mineral/element concentrations in a well-studied Mesoamerican × Andean genepool population of cultivated beans derived from the cross DOR364 × G19833; (2) determine the inheritance of the concentration of minerals/elements through recombinant inbred line (RIL) analysis; (3) evaluate the associations of QTL for various macronutrients or trace element in seed with iron and zinc concentration QTL that have been evaluated for biofortification breeding of common beans.

## Materials and Methods

### Genotypes and Experimental Design

The experiments were carried out on a population of F_9:11_ RIL genotypes from the cross DOR364 × G19833 (abbreviated D × G) as described previously (Blair et al., 2009b). The population and parents were grown across two field sites: first in Popayán, Cauca, Colombia (1,730 m above sea level, 18°C average yearly temperature, 2124 mm annual rainfall, Dystrudepts soil type, pH 5.6) and second in Darien, Valle de Cauca, Colombia (1400 m; 20°C average yearly temperature, 1650 mm annual rainfall, Udand soil type, pH 5.6). Both experiments consisted randomized complete blocks with two repetitions at each site for a total of four replicates in the experiments. Parents were planted along with the RIL lines as duplicate entries of DOR364 and G19833 giving a total of eight replicates in the experiments. Trials were managed with recommended fertilization rates for these locations (60 kg of P ha^-1^ as superphosphate) banded in the planting row and three foliar applications of zinc and boron as microelements (300 g ha^-1^ as chelates) at 14 and 21 days after planting. Preventive treatments of fungicide (Derosal at 1 L ha^-1^) and insecticide (Lorsban at 2 L ha^-1^) were used to control pests and pathogens. In both trials, plots were hand harvested and threshed to avoid contamination by metal machinery. Samples from the field repetition harvests were combined to minimize the cost of mineral analysis per location as residual heterozygosity for this population and variation in seed accumulation for iron and zinc at these sites has been shown to be low for these highly homozygous lines and for these carefully managed fields (Blair et al., 2009b). We also wanted environmental variation to be taken out of consideration in our search for and detection of QTL.

### Sampling and ICP Methodology

Samples were carefully prepared so that the seed did not come into contact with contaminants. Sample preparation consisted of 5 g of seed ground in Teflon chambers using non-chipping Zirconium balls in a Retsch mill. Samples of whole bean seeds were surface cleaned with water and then ethanol to remove soil and dust and oven dried before grinding. Samples of parents were also analyzed with two replicates per site. ICP-OES analysis (abbreviated ICP) was applied for the seed samples for all genotypes and parents from both trials using an ARL 3580 ICP-OES at the University of Adelaide. In all cases nitric/perchloric acid digested samples were used. Acid digestion involved a total of 0.25 mg of sample and 5 mL of a 2:1 mixture of 65% nitric acid (HNO_3_) and 70 % perchloric acid (HClO_4_) in a 50 ml Taylor digestion tubes for 2 h followed by a heat treatment of 200°C for 2 h and re-suspension in 25 mL of deionized water with samples read by software that comes with the ICP-OES instrumentation. Aluminum (Al) reading was used to determine if there was soil contamination.

### Data Analysis

Parental comparisons were made with a *t*-test between the ICP values for DOR364 (Mesoamerican) and G19833 (Andean) for each element using a threshold value of *P* < 0.05. Analyses of variance (ANOVA) were carried out using the program Statistix version 8.0 (Analytical Software, Tallahasse, FL, USA). QTL were detected with composite interval mapping (CIM) analysis that was carried out using the software program QTL Cartographer v. 1.21 (Basten et al., 2001) and the following parameters: 10 cM window size, 1 cM walkspeed, analysis by forward and backward multiple linear regression for each chromosomal position with a global significance level of 5% and probability thresholds of 0.05 for the partial *F* test for five significant background marker inclusion or exclusion. Determination coefficients were calculated for each interval separately (R2) and for each interval given the background markers (TR2) to determine the phenotypic variance explained by a single QTL (either alone or in conjunction with all other significant intervals). Population distributions were evaluated for normality with QTL Cartographer and LOD (log of the odds) thresholds for the individual QTL for each trait were determined by the generation of 1000 permutations of the data for that trait (Churchill and Doerge, 1994). Results were displayed using QTL Cartographer and represented graphically with standard drawing software, to designate genomic regions that proved to be significant in the analysis described above. The genetic map for the D × G population was based on the high LOD map presented in Blair et al. (2009a) where other QTL for seed Fe and Zn concentration were located as well as the same map enhanced with phytate related genes and seed phytate concentration QTL from Blair et al. (2012).

## Results

### Parental Contrasts in Seed Elements

The parental contrasts for the elements are given in **Table 1.** Significant differences were found for 13 out of the 15 elements analyzed, but only 11 are described here as two elements (Fe and Zn) were presented before (Blair et al., 2009a). Where differences were significant, the elemental concentrations were more often higher in the Andean parent, G19833, than in the Mesoamerican parent, DOR364 (6 out of 11 elements). The Mesoamerican parent was higher in the elemental concentrations for Mg, Na, Ni, and S while the Andean parent was higher or similar for the remaining elements except for Cd and Co, both heavy metals. The coefficients of variation (CV) for each element reflected the site × element interactions and were generally higher for the Mesoamerican parent, DOR364 than for the Andean parent, G19833. The values expressed as percentages were higher for Boron and Calcium than for any of the other elements and reflected soil differences for these elements. The CV for S in DOR364 seed was also high (above 25%) but not in G19833 (6.7%). All other CVs were below 20% except for Mo in ground seed of G19833. The CVs were quite low (below 10%) for Cu, K, Mn, P, and S in G19833 and K, Ni, and P in DOR364. Al was not found in the samples as the seeds were washed and soil contamination was not significant as a factor for ICP-OES mineral detection.

**Table 1:** Average element concentrations in parts per million (ppm) of the genetic mapping parents DOR364, a Mesoamerican (M) genotype, and G19833, an Andean (A) genotype and their standard deviations (SD) and coefficients of variation (CV).

Element	DOR364			G19833			Significance	A vs. M
				
	Average	*SD*	CV	Average	*SD*	CV		
B (1)	10.7	4.81	44.9	13.25	8.41	63.5	^∗^	A > M
Cu (1)	7.15	1.06	14.8	7.8	0.14	1.8	^∗^	A > M
Ca (1)	1390	1145.5	82.4	950	367.7	38.7	^∗^	A < M
Cd (1)	0.3	0	0.0	0.3	0	0	ns	A = M
Co (1)	0.65	0.07	10.9	0.65	0.07	11.2	ns	A = M
K (1)	14300	424.3	3.0	16400	1272.8	7.8	^∗^	A > M
Mg (1)	1720	339.41	19.7	1685	304.06	18.0	^∗^	A < M
Mn (1)	14.45	2.05	14.2	15.45	0.64	4.4	^∗^	A > M
Mo (1)	0.785	0.12	15.3	0.845	0.21	24.3	^∗^	A > M
Na (1)	10.25	1.06	10.4	9.65	1.91	19.8	^∗^	A < M
Ni (1)	0.95	0.07	7.4	0.9	0	0	^∗^	A < M
P (1)	3000	141.42	4.7	4500	424.26	9.4	^∗^	A > M
S (1)	2210	693	31.4	2100	141.42	6.7	^∗^	A < M


*Significance column for the ANOVA results for the DOR364 × G19833 RIL population genotypes is also given as well as the comparison of Andean vs. American parent position in the distribution.*

### Inheritance of Elemental Concentrations

**FIGURE 1:**
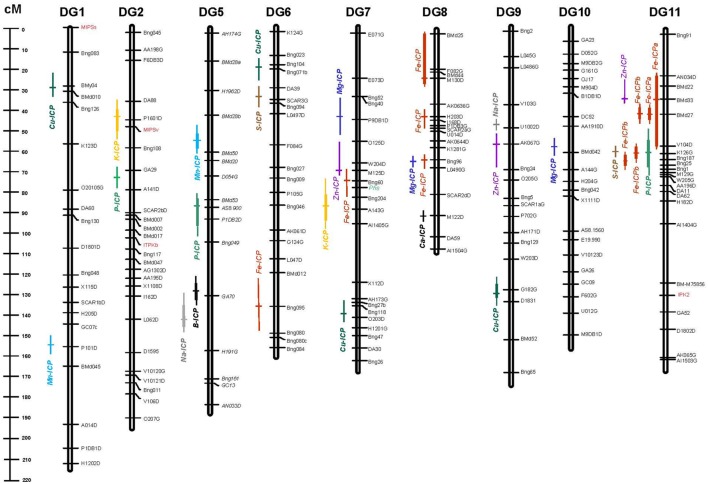
**Genetic linkage map showing quantitative trait loci (QTL) for elements detected by inductively coupled plasma (ICP) method in the DOR364 × G19833 (DG) mapping population.** QTL are shown as different colored bars for Boron (B), Calcium (Ca), Copper (Cu), Iron (Fe), Magnesium (Mg), Manganese (Mn), Phosphorus (P), Potassium (K), Sodium (Na), Sulfur (S), and Zinc (Zn) with cross marks indicating peak likelihood ratio values. Genetic markers consist of simple sequence repeat (SSR) anchor and restriction fragment length polymorphism (RFLP) markers from Blair et al. (2003, 2008, 2010a) along with genes from the phytate biosynthesis pathway (*IPK2*, *ITPKb*, *MIPSs*, and *MIPSv*) and the protein marker for phaseolin (*Phs*) highlighted in red and green text, respectively, all shown to the right of each chromosome/linkage group. Map distances between markers are shown on the scale to the left of the chromosome/linkage groups for the DG population.

Quantitative trait loci were analyzed for all of the elements with significant parental contrasts for the population. Therefore QTL were searched for 13 out of the 15 elements (two elements were not significantly different). No QTL were found for Mo or Ni even though the parents contrasted. Since Fe and Zn QTL have already been reported, in this study we only document the QTL for nine elements. In total, 21 QTL were identified for these nine elements and are described in **Table 2.** For the QTL identified, the most (four) were for Cu. Meanwhile three QTL each were found for Mg, Mn, and P. Two QTL each were found for the elements: K, Na, and S but only one QTL each was found for B and Ca. QTL locations for the individual nutrients were identified on a saturated molecular marker map for the DOR364 × G19833 population and the nearest marker to the QTL are reported in **Table 2** while the regions of the QTL are shown in **Figure 1.**

**Table 2:** Quantitative trait loci (QTL) found for various elements in the DOR364 × G19833 mapping population with their significance in Likelihood Ratio (LR), Logarithm of the ODds (LOD), determination percent (R2 and TR2) and additive values given in parts per million (ppm) elemental concentrations.

Element	Chrom. (LG)	Nearest marker	LR	LOD	R2	TR2	Source	Additive value (ppm)
B	5	GA70	17.70	3.84	16.3	33.5	DOR364	0.6
Ca	8	M122D	13.64	2.96	13.5	32.1	G19833	85.6
Cu	1	BMy04	13.30	2.89	29.1	65.4	G19833	0.8
Cu	6	BNg071b	12.09	2.63	8.0	52.4	DOR364	0.4
Cu	7	O203D	13.80	3.00	10.3	45.2	G19833	0.4
Cu	9	G182G	23.06	5.01	20.2	48	G19833	0.6
K	2	P1601D	28.00	6.08	23.2	48.9	DOR364	607
K	7	A143G	30.07	6.53	24.2	47	DOR364	584.1
Mg	7	P9DB1D	15.08	3.28	18.9	43.6	DOR364	79.6
Mg	8	BNg96	12.77	2.77	10.3	35.9	G19833	58.9
Mg	10	BNg42	15.48	3.36	12.6	35.6	G19833	65.7
Mn	1	P0101D	11.60	2.52	8.0	47.4	G19833	0.63
Mn	5	BMd50	22.11	4.80	17.0	48.3	G19833	0.91
Mn	8	L0490G	33.21	7.21	28.7	52.7	DOR364	1.2
Na	5	GA70	24.03	5.22	25.4	41.9	DOR364	2
Na	9	U1002D	11.58	2.52	9.4	33.8	G19833	1.2
P	2	GA29	13.15	2.86	8.3	50.3	DOR364	134.7
P	5	AS8.900	21.89	4.75	14.4	50.9	DOR364	182.7
P	11	K126G	29.75	6.46	20.3	50.3	DOR364	203.2
S	6	DA39	16.96	3.68	13.9	47.2	DOR364	75.3
S	11	K126G	13.31	2.89	9.3	43.7	G19833	62.2


*Source genotype refers to which parental allele provides the positive allele.*

The QTL were found on every chromosome except for two and were mostly independent of each other (**Table 2**). The Likelihood ratio (LR) values ranged from *LR* = 11.58 (equivalent *LOD* = 2.52) for the least significant QTL to *LR* = 33.21 (LOD value of 7.21) for the most significant. These QTL were for Na on chromosome 9 near marker U1002D (lowest value) and for Mn on chromosome 8 near marker L0490G (highest value). The LOD value significance in this QTL study was defined by permutation tests so thresholds varied but were confident for the declaration of the QTL found as per Asfaw and Blair (2012).

Higher LOD value QTL were identified for K, Mn, and P compared to Ca, Cu, and Mg which had mostly moderate LOD values, with the exception of one intermediate to high LOD value of 5.0 for a Cu QTL on chromosome 9 near the marker G182G. Other intermediate LOD value QTL (above 3.5) were found for B on chromosome 5 (LOD - 3.8), for Mn on chromosome 5 (*LOD* = 4.8), Na on chromosome 5 (*LOD* = 5.2) and P on chromosome 5 and chromosome 11 near markers AS8.900 (*LOD* = 4.8) and K126G (*LOD* = 6.5), respectively.

The determination values for individual markers (R2) ranged from highs of 29.1 and 28.7% (for Cu QTL on chromosome 1 and Mn QTL on chromosome 8) to low of only 8.0% (for Cu QTL on chromosome 6 and Mn QTL on chromosome 1).

Total R2 (TR2) determination values ranged from a low of 32.1% for the combined effect of the Ca QTL on chromosome 8–65.4% for the combined effect of the Cu QTL on chromosome 1 together with five next most significant background markers. Similar to even mix of elements with higher concentration in one parent or the other, the positive source allele for the QTL identified were about equally from the Andean accession genotype G19833 (10 loci) and from the Mesoamerican breeding line genotype DOR364 (11 loci). G19833 contributed the positive allele for the single QTL for Ca while DOR364 contributed the positive allele for the single QTL for B. For Cu and Mg two thirds or more of the loci had positive Andean alleles; while for Na and S the positive alleles were one half from Andean sources and one half from Mesoamerican sources. The positive allele for all K and P QTL were from DOR364; while the positive alleles for all Mn QTL were from G19833.

## Discussion

The ICP technology had a number of advantages for element evaluation in common bean. A large number of heteroelements and metals such as Ca, Mg, P, and S, or Cu, Cd, Co, Fe, Mn, Mo, Ni, and Zn (Garcia et al., 2006), respectively, could be accurately detected and quantified in the seed flour through ICP and the instrument proved to be very reliable and robust, with very low detection limits down to ppm and even ppt for certain elements (Montaser, 1998; Nelms, 2005). ICP also had high spectral resolution for non-metals as well as for minerals and tolerated the differences in the bean flour matrix from genotype to genotype in the mapping population detecting the elements regardless of their chemical matrices and overall environments (Łobiński et al., 2006). This made the ICP method useful for both parental element evaluation and QTL detection in the recombinant inbred line population.

From the first part of the study on the parental seed DOR364 and G19833 were contrasting in a large number of element concentrations and ICP was a valuable technique for detecting the content of newly evaluated elements with low concentrations in ground common bean flour, just as it had been for the Fe and Zn studies previously (Blair et al., 2009a, 2010a,b, 2011, 2013). This was due to low concentration thresholds and detection limits for ICP compared to the previous analytical methods used in common beans (Blair et al., 2009a, 2010a). The ICP method was found to produce consistent and valuable information on element concentrations for various metals such as Cu, Fe, Ni, Zn, as well as cations such as Ca and Mg and the hetero-elements B, S, and P (Ammann, 2007). The ICP method has become the technique of choice in determining elemental concentrations (Caruso et al., 2000), covering a broad field of elements, metals, metalloids, and metabolites (Hirner and Emons, 2004).

A major advantage of ICP was that it provided precise quantification via the element signal and was not affected by the matrix from which the element is derived (Łobiński et al., 2006). ICP could be used for evaluating a complex and dense plant organ such as a seed, and is also a method for analyzing food samples or grains destined for food since the method relies on a small sample. Although beyond the scope of this study, the ICP method can also be used in combinations of beans with vegetables or meat products with which they are commonly consumed. Major nutrients is of most interest since Mg is required for photosynthesis and leaf development, while K is needed for stomata and vacuole functioning. Mo is needed by legumes for nodule function. Mn together with Ca is important for plant cell wall strength and also brain development during child development (Caballero, 2002). Carbon (C) and nitrogen (N) are not analyzed by ICP and this is perhaps one disadvantage of using ICP compared with different types of mass spectroscopy or elemental analysis (Łobiński et al., 2006).

In the genetic portion of this study, we found the inheritance of seed concentration of almost all elements to be oligogenic to multigenic. We found several elements (Cd and Co) to be highly influenced by environment factors while others were less so and could be evaluated for QTL. Two elements were found in low amounts (Mo and Ni) and were not highly variable in the population, so QTL were not detected for these. The findings of quantitative inheritance for most elements are similar to the conclusions about Fe and Zn concentration in common bean as reviewed in Blair et al. (2009a).

Some of the QTL for elements evaluated in this study aligned with QTL for seed Fe and Zn concentration from a previous study in this same population (Blair et al., 2009a) or with QTL for phytate and P concentration or content in this same population (Blair et al., 2012) or in the population G2333 × G19839 (Blair et al., 2009a). Most notably, one QTL for K and one for Mg from this study were found to be in the region of the *Phs* locus on chromosome 7, an area of the genome carrying multiple genes that influenced Fe and Zn concentration (Blair et al., 2009a).

Another QTL for Mg near the marker Bng96 on chromosome 8 aligned with a previous QTL for Fe from that study as well as did a QTL for S on chromosome 11 found near a cluster of previously described QTL for Fe and Zn. One QTL for P concentration on chromosome 11 as identified by ICP in this study correlated with a previous QTL for P levels as identified by atomic absorption in the DOR364 × G19833 population (Blair et al., 2012). However, the other QTL for P identified in this study on chromosome 2 and chromosome 5 were separate from QTL for P or phytate concentration from that previous study.

These new QTL for P could be interesting for breeding purposes especially as the QTL on chromosome 5 was linked to the vegetative expressed myo-inositol phosphate synthase gene (*MIPSv*) that is basal to phytate synthesis and was mapped by Blair et al. (2012). The *MIPSv* associated QTL could therefore play a role in whole plant accumulation of P that then would be transported to the seed. Phytate may be a binding agent for some of the minerals which is why there may be a relationship between QTL. The combination of phytate or P related QTL with loci controlling Fe, Zn are important in determining the nutrient availability from bean seeds of these trace elements for cellular metabolism in humans, especially for those that are at risk of iron deficiency anemia (Moraghan et al., 2002).

Other QTL for trace elements like Cu and Mn could be important from a health perspective as their deficiency can lead to stunted growth in children (Caballero, 2002). Both minerals also play major role in reducing oxidative stress, and along with Zn, Mn, and Cu at varying levels in the diet are purported to reduce the risk of chronic diseases and age-related degenerative diseases (Steinmetz and Potter, 1996; Buring and Gaziano, 1997; Pham-Huy et al., 2008). For example the formation of superoxide dismutase requires copper (Cu), Mn, and Zn (Chen et al., 2000).

The QTL for these elements can also be important for plant physiological reasons. Notably, the balance of Mn with other positively charged ions such as K, Mg, and Na is also important for plant homeostasis, photosynthesis, stomatal activity and root function (Cheniae and Martin, 1968). Deficiency in K can be observed on some sandy soils in dry or wet environments (Clarkson and Clarkson, 1995) but is not always critical in common bean (Blair, 2013b). A response to K fertilization of common bean in Eastern Africa showed that breeding for K-use efficiency should be a goal of programs that try to improve the quality of legume seeds in the process of biofortification (Blair, 2013a). The variability in LOD scores could be due to the penetrance of the genes underlying the QTL or due to the environmental site effects as seen for the parental comparisons and this would affect the success in breeding.

Meanwhile, QTL for K could be useful if found to be associated with stomatal activity and subsequent water use efficiency or with the rate of photosynthesis through production of ATPs which in turn are associated with sugar and other nutrient (nitrates, phosphates, Ca and Mg) transport through phloem or xylem, respectively. The multiple role of K in protein and starch synthesis and in overall plant development and crop quality make the QTL for this element worthy of further study. The lack of QTL for heavy metals such as Cd, Co, Ni, and Mo could show that these are in minimal concentration on the uncontaminated soils used in our experiment. Therefore, more specific agricultural soils would be needed to determine the genetic control of heavy metals in bean seeds.

Certain other QTL could be important for nutrition and plant physiology together. For example the QTL for S concentration could indicate the presence and amount of essential sulfur-containing amino acids which are important in legumes (Iqbal et al., 2006). The QTL for B concentration could indicate tolerance to boron toxicity, although this is rarely a problem in common beans since this crop is more likely to be grown on acid soils rather than alkaline soils where high boron can be a problem (Hayes et al., 2015).

Apart from RIL analysis in common bean, the most extensive studies for uncovering loci involved in the accumulations of the elements mentioned in other legumes above have used the GWAS (genome wide association) approach. The most notable GWAS studies so far have been in the garden pea with ICP nutrient accumulation genes detected through a whole genome scan (Cheng et al., 2015). Basic studies have also been done in *Arabidopsis* seeds where a gamut of minerals was evaluated by QTL analysis (Vreugdenhil et al., 2004) and genome wide scans (Lahner et al., 2003). Similarly QTL analysis in Lotus and Medicago have been valuable in gene discovery (Francki et al., 2009; Sankaran et al., 2009).

Our study showed that common beans are good sources of many essential nutrients, beyond only Fe and Zn (Blair et al., 2009b). Apart from being rich in Ca, Cu, Fe, K, Mg, Mn, P, and Zn, common beans, like all grain legumes are usually consumed whole not milled, so their cation mineral concentration reflects their concentration in food as well (Pennington et al., 1995a,b,c). Overall, the range of Ca, Mg, P, and S in the common bean seed were higher by 10–20 fold over the amount of B, Cu, Mn, and Na, which are lower than Fe and Zn (Blair et al., 2010a,b, 2011). Legumes are useful because of the high harvest index of minerals and nutrient retention even when boiled or cooked into a dish (Wang et al., 2003; Bohra et al., 2015).

In addition, common beans are one of the legumes of greatest importance worldwide and are a staple crop for the poor in Latin America and Eastern and Southern Africa making it worthy of biofortification (Blair, 2013a). It therefore makes sense to develop common bean varieties taking into account the seed nutrient concentration and QTL controlling these traits. QTL validation is needed for all the regions detected to influence mineral accumulation in this study by repeating the experiment in new years and especially at additional sites which represent different soil nutrient profiles. From a research point of view, the genetic dissection of nutrient uptake and seed accumulation in a crop plant such as common bean is part of a movement termed “Ion Genomics” (Rea, 2003) and is a valuable starting point to understanding the genes underlying nutrient use by grain legumes.

## Author Contributions

MB wrote project, analyzed data and prepared manuscript, table and figures, XW and DB assisted in manuscript writing, editing and organization, CA provided data analysis.

## Conflict of Interest Statement

The authors declare that the research was conducted in the absence of any commercial or financial relationships that could be construed as a potential conflict of interest.
